# Live cell *in situ* lysosomal GCase activity correlates to alpha-synuclein levels in human differentiated neurons with LRRK2 and GBA1 mutations

**DOI:** 10.3389/fncel.2023.1229213

**Published:** 2023-10-16

**Authors:** Adahir Labrador-Garrido, Siying Zhong, Laura Hughes, Shikara Keshiya, Woojin S. Kim, Glenda M. Halliday, Nicolas Dzamko

**Affiliations:** School of Medical Sciences, Faculty of Medicine and Health and the Charles Perkins Centre, University of Sydney, Camperdown, NSW, Australia

**Keywords:** Parkinson's disease, GCase, alpha-synuclein, pluripotent stem cells, LRRK2

## Abstract

**Introduction:**

Heterozygous mutations in *GBA1*, which encodes the lysosomal hydrolase glucocerebrosidase (GCase), are a common risk factor for the neurodegenerative movement disorder Parkinson's disease (PD). Consequently, therapeutic options targeting the GCase enzyme are in development. An important aspect of this development is determining the effect of potential modifying compounds on GCase activity, which can be complicated by the different methods and substrate probes that are commonly employed for this purpose.

**Methods:**

In this study, we employed the GCase substrate probe 5-(pentafluorobenzoylamino)fluorescein di-D-glucopyranoside (PFB-FDGlu) in combination with live cell imaging to measure GCase activity *in situ* in the lysosome.

**Results:**

The live cell assay was validated using the GCase inhibitor conduritol-B-epoxide and with *GBA1* knockout neural cells and was then used to assess GCase activity in iPSC differentiated into neural stem cells and neurons that were obtained from idiopathic PD patients and PD patients with the LRRK2 G2019S and GBA N370S mutations, as well as controls (*n* = 4 per group). Heterogeneity in GCase activity was observed across all groups. However, a significant inverse correlation between GCase activity and levels of alpha-synuclein protein was observed.

**Discussion:**

The live cell imaging assay for GCase activity could be useful for further understanding the role of GCase in PD and screening potential modifying compounds in differentiated human cell models.

## Introduction

Variants in the lysosomal hydrolase glucocerebrosidase (GCase), encoded by *GBA1*, may be associated with up to 15% of cases of the neurodegenerative movement disorder Parkinson's disease (PD) (Smith and Schapira, 2022). At least 300 mutations have been described across *GBA1*, and a number of these mutations are associated with reduced GCase activity (Hruska et al., 2008). The exact mechanism by which GCase contributes to PD remains to be elucidated, but likely mechanistic candidates are reduced GCase activity and/or imbalances in the GCase substrates. The former can lead to lysosomal dysfunction and impaired clearance of the hallmark pathological PD protein alpha-synuclein (Mazzulli et al., 2016a), while imbalances in the GCase substrate, glucosylceramide (GlcCer), can trigger inflammation (Pandey et al., 2017). Consequently, small-molecule activators and/or stabilizers of GCase activity have emerged as potential PD therapeutics (reviewed in Ryan et al., 2019; Gegg et al., 2022; Martínez-Bailén et al., 2022).

After synthesis in the endoplasmic reticulum, GCase is trafficked to the lysosome in association with lysosomal integral membrane 2 (LIMP2) (Reczek et al., 2007). In the lysosome, GCase is stabilized and activated via interaction with saposin C (SapC) and proceeds with its enzymatic activity to hydrolyse GlcCer into glucose and ceramide at lysosomal pH (Tamargo et al., 2012). A number of methods have been established to assess GCase activity. However, some of these methods require lysis of cells and subsequent assays of GCase activity disassociated from lysosomal pH and saposin C and do not allow for single or live cell analysis of GCase activity (Ysselstein et al., 2021).

One way to assess the activity of GCase *in situ* in the lysosome in cellular models is with the selective GCase substrate 5-(pentafluorobenzoylamino)fluorescein di-D-glucopyranoside (PFB-FDGlu). The PFB-FDGlu probe enters the cell via pinocytosis and travels to the lysosome, where it is metabolized by GCase to release fluorescein (Lorincz et al., 1997). The fluorescence intensity of fluorescein as measured by flow cytometry has been used to assess GCase activity in peripheral immune cells of patients with Gaucher disease (Rudensky et al., 2003), a lysosomal storage disease caused by homozygous mutations in *GBA1* (Hruska et al., 2008; Riboldi and Di Fonzo, 2019), as well as to demonstrate that GCase activity is reduced in monocytes from patients with PD (Atashrazm et al., 2018; Hughes et al., 2021; Wallings et al., 2022). However, this flow cytometry method is suboptimal for mechanistic studies and therapeutic screening assays in human neurons, as human neurons are highly sensitive to the oxidative and shear stresses that occur with flow cytometry and rapidly die.

To assess *in situ* lysosomal GCase activity in human neurons, specifically neurons differentiated from induced pluripotent stem cells (iPSC), the PFB-FDGlu GCase assay was modified in this study to work with live cell imaging instead of flow cytometry. We demonstrated the specificity of the assay using the GCase inhibitor conduritol b-epoxide and *GBA1* knockout murine neural cells and assessed GCase activity in neural stem cells and neurons differentiated from iPSC obtained from patients with PD-associated mutations LRRK2 G2019S and GBA1 N370S, as well as iPSC from PD patients without these mutations and from controls. Heterogeneity in GCase activity was evident across the cell lines, but a significant inverse correlation between GCase activity and alpha-synuclein levels was measured in the differentiated neurons. The live cell imaging protocol using PFB-FDGlu could, therefore, be useful for understanding how GCase activity contributes to PD pathogenesis and/or screening for modulators of GCase activity in neural cells *in vitro*, and results from the differentiated neurons add to the evidence that GCase contributes to the regulation of alpha-synuclein turnover.

## Methods

### SH-SY5Y and GBA1 knockout cells

Human neuroblastoma SH-SY5Y cells were maintained in DMEM/F-12 supplemented with 10% low-endotoxin fetal bovine serum, 2 mM L-glutamine, and 1% penicillin-streptomycin (all from Gibco, Thermo Fisher Scientific, USA). For experiments, cells were plated on clear bottom 96-well plates (Phenoplate, Perkin Elmer, USA) at 6 × 10^4^ cells/well and maintained as described in a previous study (Westbroek et al., 2016) for 24 h to allow the cells to attach. WT and *GBA1* knockout (KO) immortalized neural cells were kindly provided by Ellen Sidransky from the National Institutes of Health, and the generation and characterization of these cells have been described previously (Westbroek et al., 2016). These cells were cultured in neurobasal growth media supplemented with 2% B27 supplement, 1 mM L-glutamine, and 100 U/mL penicillin-streptomycin (all from Thermo Fisher Scientific, USA) in T75 flasks (Thermo Fisher Scientific, USA) pre-coated with poly-L-lysine (Sigma-Aldrich, USA) at 37°C with 5% CO_2_. For live cell imaging experiments, 2.5 × 10^4^ cells per well were plated on clear bottom 96-well plates (PhenoPlate, PerkinElmer, USA) pre-coated with poly-L-lysine and incubated at 37°C with 5% CO_2_ until confluent.

### Induced pluripotent stem cell lines

Induced pluripotent stem cell lines (iPSC) from PD patients with and without a heterozygous *GBA1* N370S mutation and matched controls were obtained from the Golub Capital iPSC Parkinson's Progression Marker Initiative (PPMI) sub-study (www.ppmi-info.org/cell-lines). The investigators within PPMI contributed to the design and implementation of PPMI and/or provided data and collected samples but did not participate in the analysis or writing of this report. For up-to-date information on the PPMI study, please visit www.ppmi-info.org. The heterozygous LRRK2 G2019S iPSC lines, tone control iPSC line, and one idiopathic PD iPSC line were generated in-house by reprogramming fibroblasts obtained from the Coriell Cell Repository, and the generation and characterization of all these lines have been described previously (Dzamko et al., 2017; Zhao et al., 2020; Chedid et al., 2022). Cell line details and available associated demographic data are provided in Table 1. IPSCs were adapted to grow on Geltrex substrate with Essential 8 media (both from Thermo Fisher Scientific, USA) and were manually passaged under a stereomicroscope, as we described previously (Dzamko et al., 2017; Zhao et al., 2020). Only early passage cells were used for experiments. The presence of GCase N370S and LRRK2 G2019S mutations, as well as the absence of these mutations from the control and idiopathic PD lines, was confirmed using Sanger sequencing. All work with human iPSC was approved by the University of Sydney Human Research Ethics Committee (2017/094).

**Table 1:** Demographic data that accompanied cell lines used for this study.

**Cell line no**.	**Age**	**Sex**
7428	Control-1	72	F
7521	Control-2	67	F
4035	Control-3	65	M
12822	Control-4	65	M
15733	iPD-1	60	M
472	iPD-2	59	F
10106	iPD-3	61	F
ND29494^*^	iPD-4	80	M
ND34810^*^	LRRK2 G2019S-1	72	M
ND38262^*^	LRRK2 G2019S-2	60	M
ND30244^*^	LRRK2 G2019S-3	73	M
ND34198^*^	LRRK2 G2019S-4	58	M
17880	GBA1 N370S-1	62	M
5484	GBA1 N370S-2	59	F
6745	GBA1 N370S-3	62	M
21138	GBA1 N370S-4	61	F

IPS cells were obtained from PPMI or reprogrammed from fibroblasts obtained from the Coriell Cell repository (with the latter indicated with^*^).

### Differentiation of iPSCs from neural stem cells and neurons

Neural stem cells (NSCs) were differentiated from the iPSCs using the Gibco™ PSC Neural Induction Medium kit (Thermo Fisher Scientific, USA) as per the manufacturer's instructions. NSCs were plated on Geltrex™ LDEV-Free Reduced Growth Factor Basement Membrane Matrix (Thermo Fisher Scientific, USA) and cultured in neural expansion medium composed of 50% advanced DMEM and 50% neurobasal medium supplemented with neural induction supplement and 1X penicillin-streptomycin (all from Gibco, Thermo Fisher Scientific, USA). The medium was changed every 2 days. NSCs were detached with Accutase (Thermo Fisher Scientific, USA) and passaged in the presence of 10 μL/ml RevitaCell supplement (Thermo Fisher Scientific, USA) when they reached 80% confluence. For experiments, NSCs were plated on clear bottom 96-well plates (PhenoPlate, PerkinElmer, USA) coated with Gletrex (Thermo Fisher Scientific, USA) at 2 × 10^4^ cells/well and maintained as described for 24 h to allow the cells to attach. For neuronal differentiation, NSCs were plated on clear bottom 96-well plates (PhenoPlate, PerkinElmer, USA) coated with 10 mg/mL poly-L-ornithine (Sigma-Aldrich, USA) and 10 μg/mL laminin (Thermo Fisher Scientific, USA) at a density of 1 × 10^4^ cells/well. NSCs were then differentiated into neurons over 21 days using neurobasal plus medium supplemented with 2% B-27 serum-free supplement, 2 mM L-glutamine, and 1% penicillin-streptomycin (all from Gibco, Thermo Fisher Scientific, USA), as we have described previously (Dzamko et al., 2017; Zhao et al., 2020; Chedid et al., 2022). Half of the differentiation medium was replaced every second day until cells were either fixed for confocal imaging or treated for live cell GCase activity assay measurement.

### Cell viability

Lactate dehydrogenase (LDH) present in the supernatant was measured using the CytoTox96 non-radioactive cytotoxicity assay (Promega Corporation, USA) following manufacturer instructions before and 2 h after imaging for GCase activity. A 1% Triton-X100 (Sigma-Aldrich, USA) solution was used as a positive control for maximum LDH release. Cell survival percentage was calculated as (sample LDH release (OD_490_)/maximum LDH release (OD_490_))^*^100.

### Immunocytochemistry

Cells were washed with 1 X DPBS (Gibco, Thermo Fisher Scientific, USA) and fixed with 4% paraformaldehyde at room temperature for 15 min. Cells were then washed twice with 1 X PBS and permeabilised with 0.3% Triton X100 (Sigma-Aldrich, USA) in 1 X PBS for 15 min, followed by blocking with 3% bovine serum albumin in 1 X PBS at room temperature for 1 h. Incubation with primary antibodies [rabbit polyclonal MAP2 (Abcam, 32454; 1:300), mouse monoclonal Nestin (ab6320, 1:500), and mouse monoclonal alpha-synuclein (BD Biosciences, 610787; 1:200)] was performed in blocking buffer at 4°C overnight. We previously demonstrated the specificity of the alpha-synuclein antibody using alpha-synuclein knockout SH-SY5Y cells (Gao et al., 2019). The wells were then carefully washed three times with 1 X PBS and incubated for 1 h at room temperature with appropriate secondary antibodies: donkey anti-mouse Alexa Fluor 488/555 and donkey anti-rabbit Alexa Fluor 488 (Life Technologies 1:500) diluted in blocking buffer. The wells were again washed four times with 1 X PBS, and DAPI #D1306 (Thermo Fisher Scientific, USA), 300 nM, was added in the third wash to counterstain the nuclei. The wells were left in the fourth PBS wash and imaged soon after.

### Confocal imaging and analysis

Stained wells were imaged using a Nikon A1R Advanced Confocal Microscope. Images were captured at 40X magnification. At least three images were taken and well analyzed. Three independent wells were stained in three independent experiments per cell line. Nestin and MAP2 were analyzed by manually counting the number of nuclei positive for the corresponding marker. Alpha-synuclein was measured using the image analysis software Fiji (Schindelin et al., 2012). For every image, nuclei were counted manually, and then, the flourescent signal was thresholded using unstained controls to select the minimum value, and the mean fluorescence intensity (MFI) was detected per image and divided by the number of nuclei to obtain the MFI of alpha-synuclein/cell.

### Opera Phenix live cell GCase assay

Designated wells were first treated with indicated concentrations of the GCase-specific inhibitor Conduritol B epoxide (CBE) (Sigma-Aldrich #C5424, USA), 400 nM of Bafilomycin (Sigma-Aldrich #B1793, USA), or an equivalent volume of DMSO for 1 or 4 h for bafilomycin. Following this, 20 nM Hoesch33342 (Bio-Rad PUREBLU™ #1351304) and 75 mM GCase substrate PFB-FDGlu [5(Pentafluorobenzoylamino) Fluorescein Di-β-D-Glucopyranoside] (Thermo Fisher Scientific #P11947, USA) were added. Plates were then rapidly inserted in the high-content imaging system Opera Phenix Plus (Perkin Elmer, USA) and imaged at the indicated times. Images were taken using a 20X water immersion objective, and at least 8 fields of view were imaged per well. Every experiment was performed in triplicate, with three wells per condition each time. Images were analyzed using Harmony software following standard protocols. Briefly, nuclei were detected and counted using the inbuilt “Detect nuclei” feature; the green fluorescence signal was thresholded using untreated wells (no CBE and no PFB-FDGlu treatment, only Hoechst added) to select the threshold value; and the MFI was calculated per field of view and divided by the number of nuclei present in the image to obtain MFI/cell values. The GCase activity ratio was calculated as follows: (MFI/cell of PFB-FDGlu-treated wells divided by the MFI/cell of PFB-FDGlu+CBE-treated wells). For colocalization experiments, designated wells were stained with Lysotracker Deep Red (Thermo Fisher Scientific #L12492, USA) at 75 nM for 45 min to allow the reagent to enter the cells and stain the lysosomal compartment. Following this, the medium was replaced with fresh media containing 20 nM Hoesch33342 and 75 mM PFB-FDGlu. Plates were then imaged at 30 min at 40X water immersion objective.

### Statistical analysis

Statistical analysis was performed using GraphPad Prism version 9.0 for macOS. A one-way ANOVA with Bonferroni *post-hoc* testing and adjusted *p*-values for multiple comparisons was used to compare effects between groups or individual cell lines. Significance was accepted at a *p*-value of < 0.05. A Spearman's correlation was performed to determine the association between GCase activity and alpha-synuclein.

## Results

### Optimization of a live cell GCase assay

**Figure 1:**
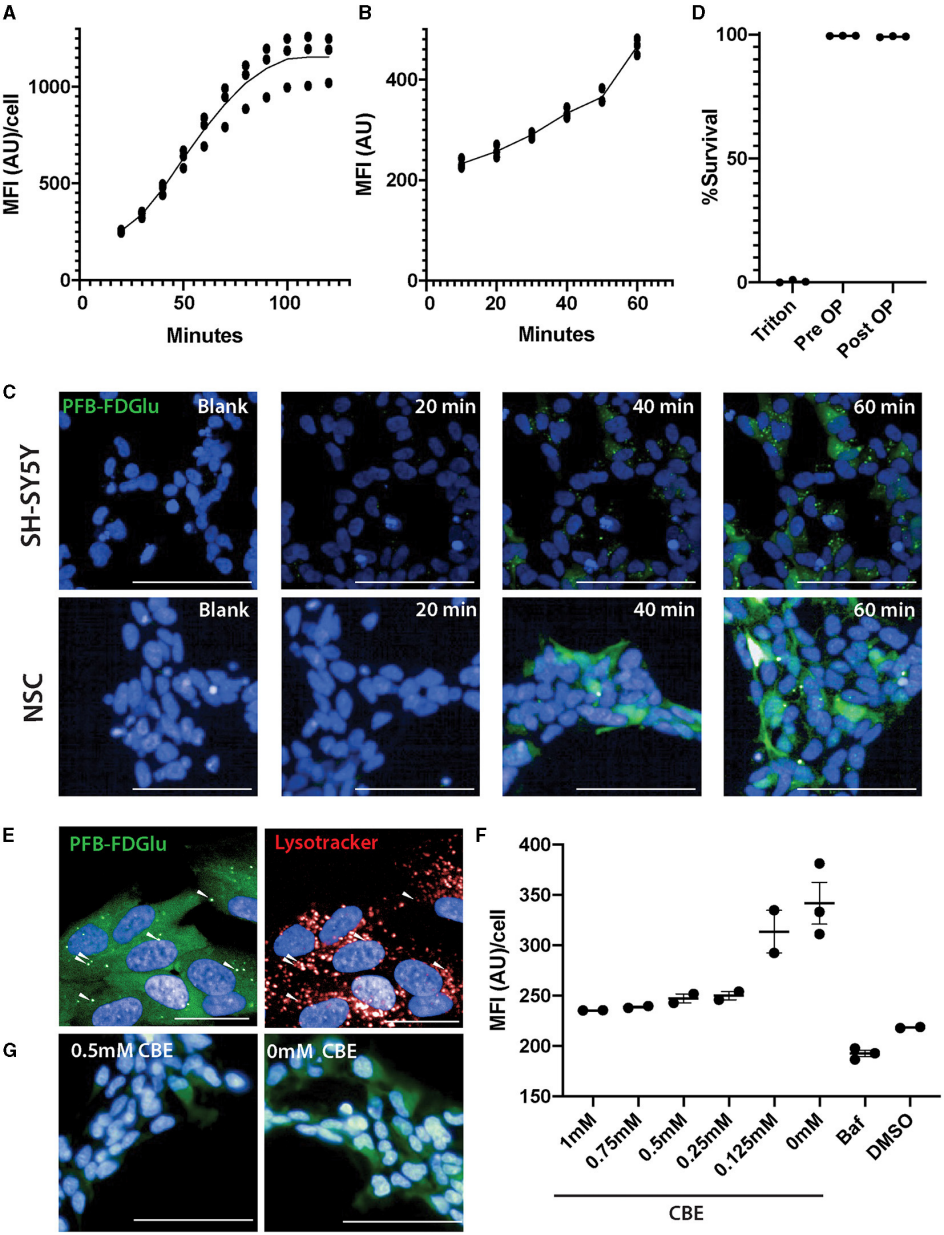
Optimization of GCase live cell assay in SH-SY5Y and neural stem cells (NSCs). **(A)** Fluorescence from the metabolism of the GCase substrate PFB-FDGlu was measured every 10 min for 2 h in SH-SY5Y cells and for 1 h in NSCs from a control line **(B)**. **(C)** Representative images of the PFD-FBGlu fluorescence of blank (no PFB-FDGlu treated, representative image at 60 min) and treated wells at 20, 40, and 60 min in SHSY5Y (top row of images) and NSCs (lower row of images). **(D)** Cell viability measurement based on lactate dehydrogenase release before and after 2 h of imaging inside the Opera Phenix. **(E)** Representative images of the colocalization of PFB-FDGlu fluorescence puncta with Lysotracker puncta and diffuse PFB-FDGlu cytoplasmic staining. **(F)** CBE dose-dependent response and bafilomycin treatment on the effect of PFB-FDGlu fluorescence with horizontal bars representing the mean of each condition. **(G)** Representative images of the PFB-FDGlu fluorescence of 0.5 and 0 mM CBE at 45 min. **(G)** Representative images of the colocalization of PFB-FDGlu fluorescence puncta with Lysotracker puncta and diffuse cytoplasmic staining. Dots on graphs represent the MFI/cell on an independent well (*n* = 2–3); white scale bar for **(D, F)** = 100 μm; white scale bar for **(G)** = 25 μm. MFI/cell, mean fluorescence intensity per cell, represented in arbitrary units. Results are representative of at least 2 independent experiments.

**Figure 2:**
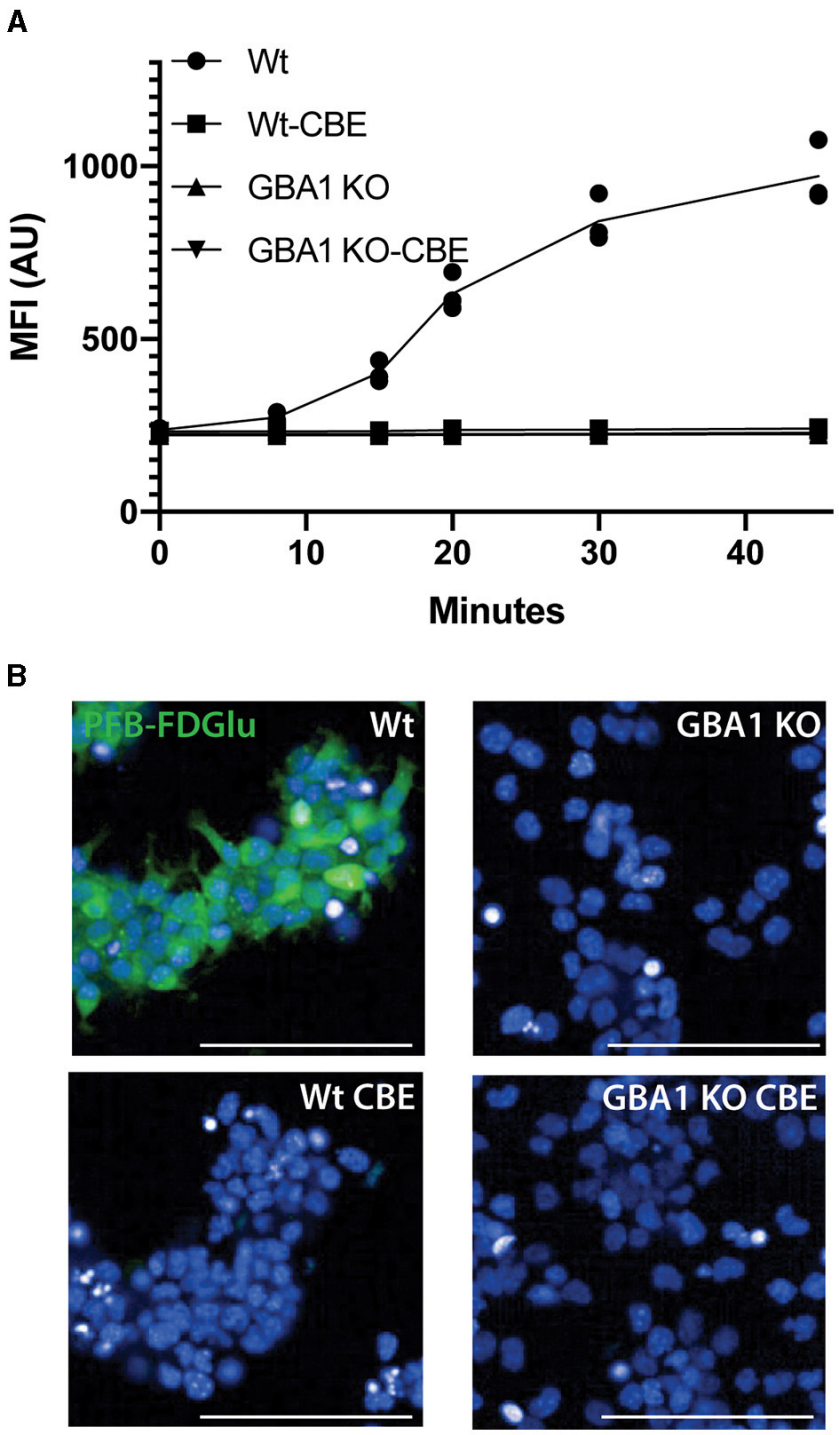
GCase live cell assay validation in *GBA1* KO cell line. **(A)** PFB-FDGlu fluorescence was measured over time in Wt and GBA1 KO neural cell lines in the presence or absence of CBE. **(B)** Representative images of the PFB-FDGlu fluorescence at 45 min in wildtype and *GBA1* KO cell lines without CBE. Each dot represents the MFI/cell on an independent well (*n* = 3); white scale bar = 100μm; MFI/cell, mean fluorescence intensity per cell, represented in arbitrary units.

Initial attempts to assess GCase activity in neurons differentiated from IPSC resulted in reduced cell viability (75 ± 3%, *n* = 8), prompting the consideration of live imaging where the cells would not need to be detached and centrifuged. Initial time course experiments in differentiated SH-SY5Y cells (Figure 1A; Supplementary Video 1) and a control neural stem cell line (Figures 1B, C) indicated that the fluorescence intensity of PFB-FDGlu following its metabolism by GCase could be measured in these cells over a linear range of 0–60 min. In the SH-SY5Y cells, the number of cells detected for imaging was not significantly different over the time course (6,845 ± 624 cells at baseline and 7,046 ± 701 cells at 2 h), suggesting that viability was maintained. A lack of lactate dehydrogenase in tissue culture media also indicated high cell viability during the imaging protocol (Figure 1D). Considering the fluorescent signal resulting from PFB-FDglu metabolism co-localized with lysosomes, as indicated by lysotracker staining (Figure 1E), but consistent with a previous report (Deen et al., 2022), the signal was not fully retained in lysosomes and diffused over time to give cytoplasmic staining. Importantly, the PFB-FDGlu signal was blocked in a dose-dependent manner with the GCase inhibitor CBE (Figures 1F, G) and the lysosomal inhibitor bafilomycin (Figure 1F). From these initial experiments, a time point of 45 min and a concentration of 0.5 mM CBE were selected for further measurements. To further confirm the specificity for lysosomal GCase, we also measured GCase activity in WT and *GBA1* KO mouse neuronal-like cells. The marked reduction in PFB-FDGlu signal observed in the *GBA1* KO cells further demonstrated that the live cell assay was specific for *in situ* lysosomal GCase activity (Figures 2A, B).

### GCase activity in neural stem cells with different PD mutations

**Figure 3:**
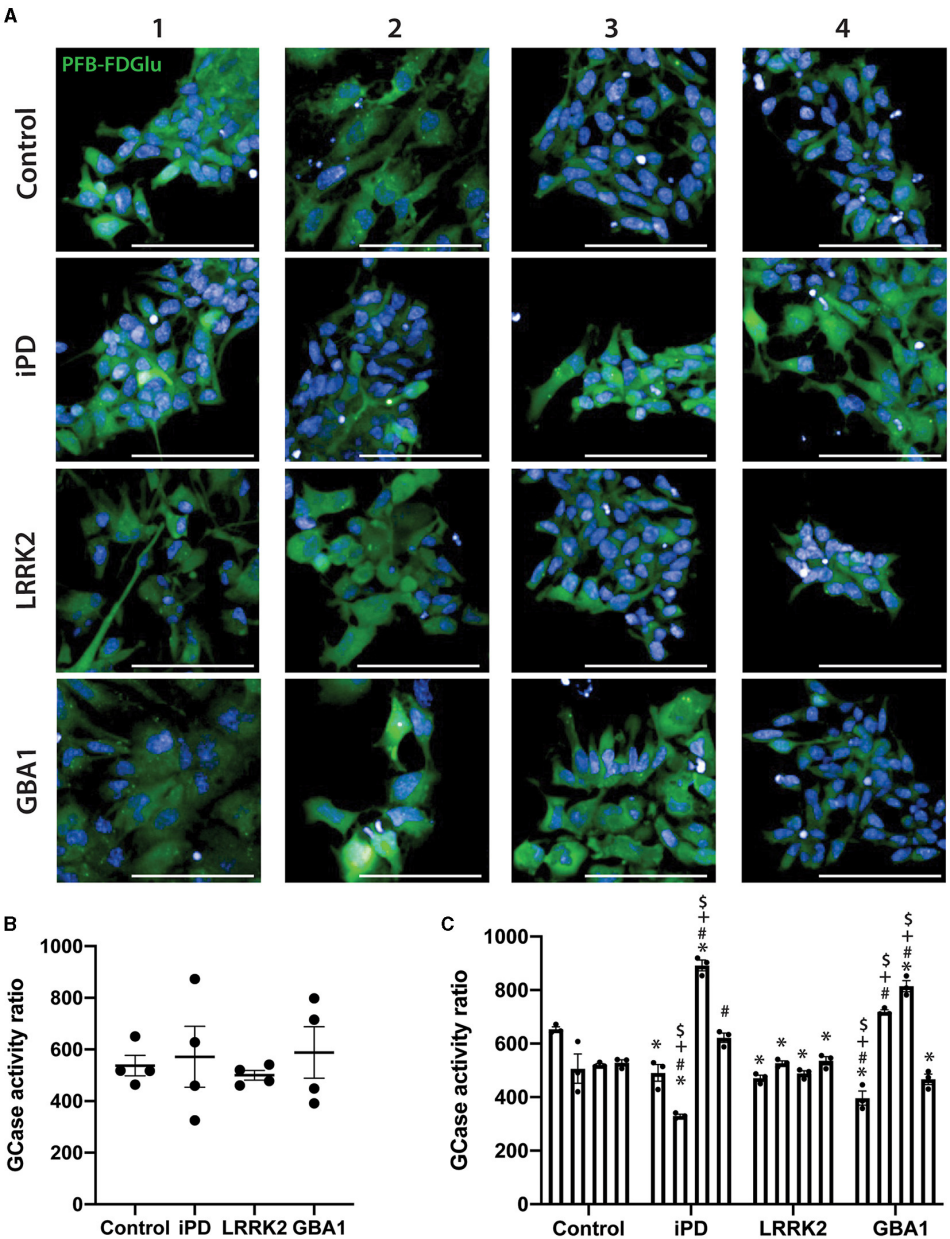
GCase activity in neural stem cells (NSCs) with different PD mutations. **(A)** Representative images of the PFB-FDGlu fluorescence at 45 min in 16 NSC cell lines. Each row represents an experimental group (see image left labels), and each column represents individual 1–4 of each experimental group. **(B)** GCase activity ratio was calculated for 16 NSC cell lines grouped by experimental group. Each dot represents the GCase activity of each cell line, calculated as the mean of 3 independent experiments; each horizontal line represents the mean of the group, and the error bars represent ± the SEM. **(C)** GCase activity ratio was calculated for all 16 NSC cell lines; each bar represents the mean GCase activity ratio of 3 independent experiments (represented as individual dots), and the error bars represent ± the SEM. *White scale bar = 100 μm; GCase activity ratio was calculated as follows (MFI/cell of PFB-FDGlu treated wells divided by the MFI/cell of PFB-FDGlu+CBE treated wells). One-way ANOVA with Bonferroni *post-hoc* testing and adjusted *p*-values for multiple comparisons was used to compare effects between groups or individual cell lines *p < 0.05 compared to the Control-1 line, ^#^*p* < 0.05 compared to the Control-2 line, ^+^*p* < 0.05 compared to Control-3 line, ^$^*p* < 0.05 compared to Control-4 line.

Nestin staining was first used to confirm the successful differentiation of the 16 iPSC lines into NSC. All lines exhibited >95% nestin-positive cells with no difference in differentiation observed with iPD, the GCase N370S, or LRRK2 G2019S mutations (Supplementary Figure S1). GCase activity was then assessed in the same run for all 16 cell lines. Representative images of GCase fluorescence for all NSC lines are shown in Figure 3A. At a group level, there was no significant difference between the control, PD, or mutation groups (Figure 3B). However, it was obvious that there was substantial heterogeneity across the different NSC lines. This was evident when cells were analyzed as their own individual lines, with a one-way ANOVA identifying a number of significant differences between lines (*p* < 0.0001). *Post-hoc* testing correcting for multiple comparisons showed that iPD-2 and iPD-3 lines were significantly lower and higher than all control lines, respectively. GBA1-1 and GBA1-3 lines were significantly lower and higher than all control lines, respectively. All LRRK2 lines were similar to control lines, except for Control-1, which was higher than all LRRK2 lines and all other control lines (Figure 3C).

### GCase activity in differentiated neurons with different PD mutations

**Figure 4:**
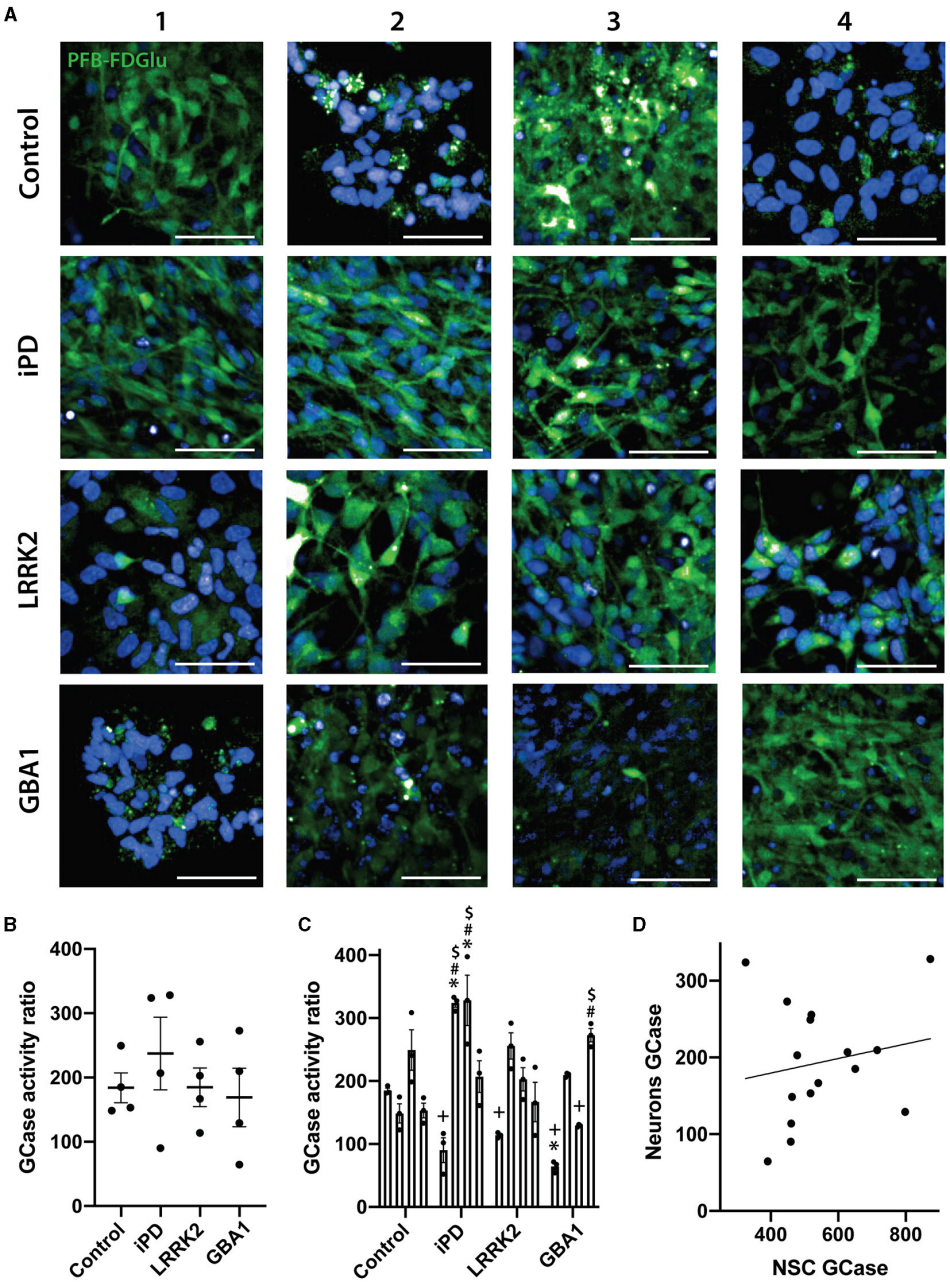
GCase activity in differentiated neurons with different PD mutations. **(A)** Representative images of the PFB-FDGlu fluorescence at 45 min in 16 neuronal differentiated cell lines. Each row represents an experimental group (see image left labels), and each column represents individual 1–4 of each experimental group. **(B)** GCase activity ratio of 16 neuronal differentiated cell lines grouped by experimental group, each dot represents the GCase activity of each cell line, calculated as the mean of 3 independent experiments, each horizontal line represents the mean of the group, the error bars represent ± the SEM. **(C)** GCase activity ratio of all 16 neuronal differentiated cell lines, each bar represents the mean GCase activity ratio of 3 independent experiments (represented as individual dots), and the error bars represent ± the SEM. **(D)** Correlation graph between GCase activity ratio on NSC vs. its corresponding differentiated neuronal differentiated culture for all 16 cell lines. Each value for the X axis represents the mean of the GCase activity ratio in NSCs of 3 independent experiments, and each value for the Y axis represents the mean of the GCase activity ratio in differentiated neurons of 3 independent experiments. *White scale bar = 50 μm; GCase activity ratio was calculated as follows (MFI/cell of PFB-FDGlu treated wells divided by the MFI/cell of PFB-FDGlu+CBE treated wells). One-way ANOVA with Bonferroni *post-hoc* testing and adjusted *p*-values for multiple comparisons was used to compare effects between groups or individual cell lines *p < 0.05 compared to the Control-1 line, ^#^*p* < 0.05 compared to the Control-2 line, ^+^*p* < 0.05 compared to Control-3 line, ^$^*p* < 0.05 compared to Control-4 line. Results are representative of at least 2 independent experiments.

We then assessed GCase activity in the same lines, but this time differentiated to neurons. MAP2 and TUJ1 staining were first used to confirm the successful differentiation of the 16 iPSC lines into neurons. All lines exhibited >95% MAP2/TUJ1 positive cells, with no difference in differentiation observed with iPD, the GCase N370S, or the LRRK2 G2019S mutations (Supplementary Figure S2). Representative images of GCase fluorescence for all differentiated lines are shown in Figure 4A. Similar to the results with NSCs, there was no significant difference between the lines in a group analysis (Figure 4B), with heterogeneity again evident. Again, when analyzed as individual cell lines, there were significant differences between lines (*p* < 0.0001). The cell lines iPD-2 and iPD-3 were significantly higher than all control cell lines except Control-3. The GBA1-1 cell line was significantly lower than Control-1 and Control-3. The LRRK2-2 line was significantly higher than the Control-2 and Control-4 lines (Figure 4C). Interestingly, there was no correlation between GCase activity assessed in the NSC and the differentiated neurons (rho = 0.15, *p* = 0.579) (Figure 4D).

### GCase activity correlates to alpha-synuclein levels in differentiated neurons

**Figure 5:**
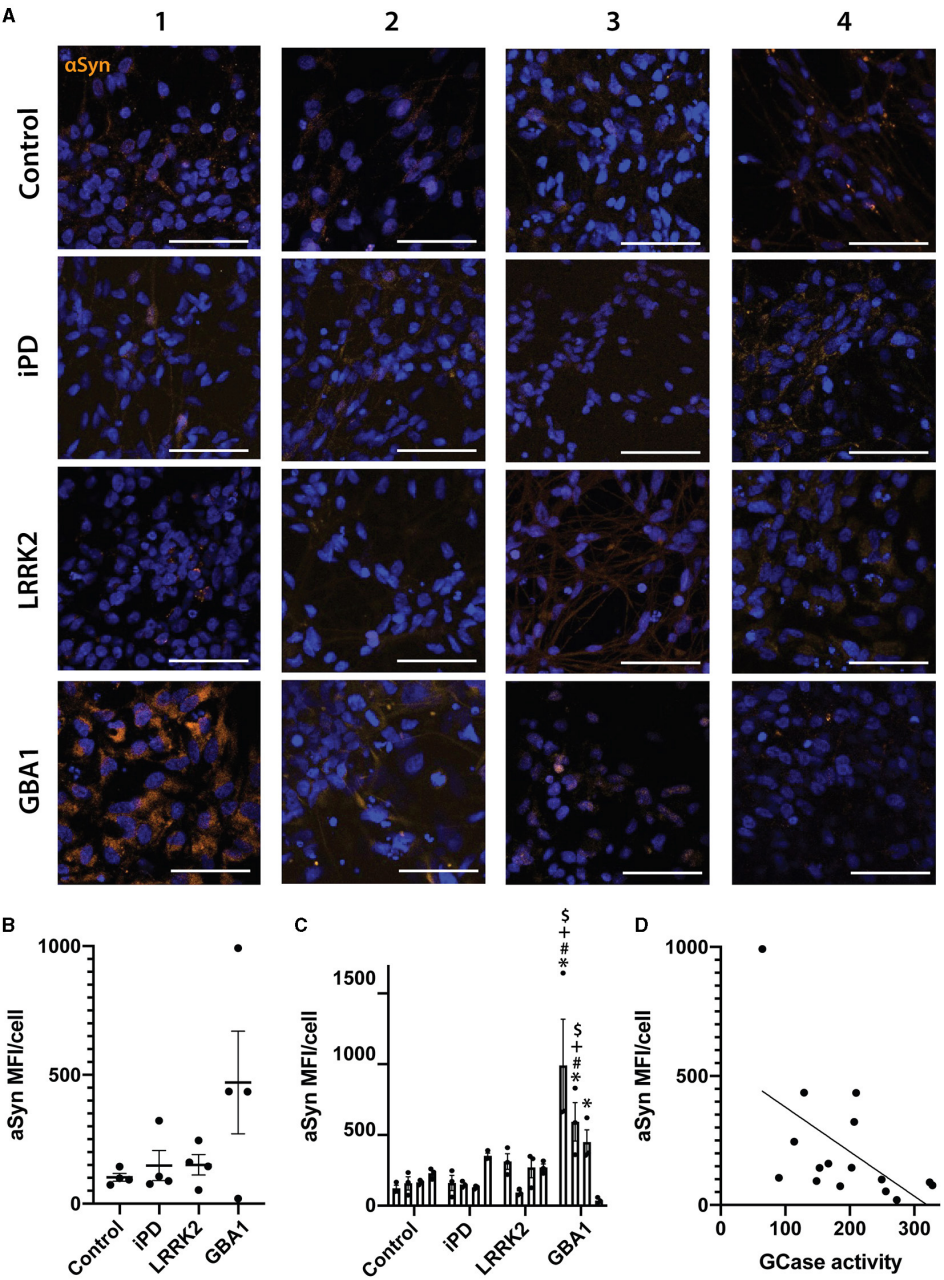
Alpha-synuclein levels in differentiated neurons correlate with their GCase activity ratio. **(A)** Representative images of alpha-synuclein immunofluorescence staining in 16 neuronal differentiated cell lines. Each row represents an experimental group (see image left labels), and each column represents individual 1–4 of each experimental group. **(B)** Alpha-synuclein MFI/cell of 16 neuronal differentiated cell lines grouped by experimental group, each dot represents the MFI/cell of each cell line, calculated as the mean of 3 independent experiments, each horizontal line represents the mean of the group, the error bars represent ± the SEM. **(C)** Individual alpha-synuclein MFI/cell of all 16 neuronal differentiated cell lines, each bar represents the mean MFI/cell of 3 independent experiments (represented as individual dots), and the error bars represent ± the SEM. **(D)** Spearman correlation graph between GCase activity ratio vs. alpha-synuclein MFI/cell for all 16 neuronal differentiated cell lines. Each value for the X axis represents the mean of the GCase activity ratio in differentiated neurons of 3 independent experiments, and each value for the Y axis represents the mean of the alpha-synuclein MFI/cell in differentiated neurons of 3 independent experiments. One-way ANOVA with Bonferroni *post-hoc* testing and adjusted *p*-values for multiple comparisons was used to compare effects between groups or individual cell lines *White scale bar = 100 μm. *p < 0.05 compared to Control-1 line, ^#^*p* < 0.05 compared to Control-2 line, ^+^*p* < 0.05 compared to Control-3 line, ^$^*p* < 0.05 compared to Control-4 line.

Finally, we also assessed alpha-synuclein levels across all 16 cell lines using immunocytochemistry (Figure 5A). Consistent with previous results, there was no significant difference when analyzed at a group level (Figure 5B), but when analyzed as individual cell lines, the GBA1-1 and GBA1-2 lines had significantly higher levels of alpha-synuclein than all control lines (Figure 5C). Interestingly, despite the heterogeneity across cell lines, there was a significant inverse correlation between GCase activity and alpha-synuclein levels in the differentiated neurons (rho = −0.54, *p* = 0.029) (Figure 5D). This may suggest that GCase activity influences alpha-synuclein levels, regardless of mutation status.

## Discussion

As mutations in *GBA1* comprise one of the strongest genetic risk factors for PD, there is much interest in understanding how mutations affect GCase function and how this enzyme could be therapeutically targeted. Fluorescent GCase substrate probes such as PFB-FDGlu (Mazzulli et al., 2016b) and the recently described LysoFQ-GBA (Deen et al., 2022) have emerged as potentially important tools, allowing for the assessment of GCase activity directly at its biological site of action, the lysosome, where lysosomal pH and interaction with SapC result in a fully active hydrolase. Combining these probes with high-content live imaging systems can facilitate studies aimed at systematically assessing the effect of *GBA1* mutations on GCase activity and/or screening for modulators of GCase activity, with particular utility for assessing GCase activity in differentiated neuronal cell lines.

A number of studies support a link between GCase activity and levels of the pathological PD protein alpha-synuclein. GCase activity is lower in postmortem brain tissues from PD patients, including idiopathic patients without *GBA1* mutations, and is associated with alpha-synuclein pathology (Gegg et al., 2012; Murphy et al., 2014; Moors et al., 2019). Studies using cells to introduce pathogenic GBA1 mutations or modulate GCase activity through knockdown or inhibition have shown increased levels of alpha-synuclein (Bae et al., 2014; Papadopoulos et al., 2018; Maor et al., 2019; Navarro-Romero et al., 2022). Furthermore, increased alpha-synuclein pathology is observed in mouse models with GCase dysfunction (Migdalska-Richards et al., 2020; Polinski et al., 2022). Exactly how GCase activity causes the accumulation of alpha-synuclein remains to be determined, but it likely involves the impairment of autophagy pathways, in particular chaperone-mediated autophagy (Kuo et al., 2022). Moreover, alpha-synuclein may also directly impact GCase activity, causing a pathogenic bi-directional loop (Mazzulli et al., 2011). However, a direct relationship between GCase activity and alpha-synuclein pathology is not always observed (Dermentzaki et al., 2013; Tayebi et al., 2017; Henderson et al., 2020; Johnson et al., 2021; Polinski et al., 2021), with different methods used for measuring GCase activity, pathological alpha-synuclein, and autophagy-lysosomal function between models contributing to complexity in the interpretation of results. In the current study, we found an inverse correlation between levels of monomeric alpha-synuclein and lysosomal GCase activity, supporting a relationship between GCase activity and alpha-synuclein turnover; however, further research is required to determine the extent to which this is due to autophagy-lysosomal dysfunction. It will also be important to determine the relationship between alpha-synuclein and GCase under pathological conditions where levels of alpha-synuclein are more robustly elevated. Indeed, the live cell imaging platform used in this study could be ideal for assessing dynamic measures of autophagy in conjunction with GCase activity in future studies, including the addition of a lysosomal marker to assess GCase colocalization with lysosomes.

Although an inverse correlation between alpha-synuclein and GCase activity was observed across all cell lines, it is also noteworthy that significant group effects due to the LRRK2 G2019S or GCase N370S mutations were not observed. Studies on heterozygous GCase N370S mutation iPSC-derived neurons have been performed before with mixed outcomes. In one study of *n* = 3 GCase N370S iPSC differentiated to dopaminergic neurons, overall levels of GCase protein were the same as control. However, there was a shift to a higher-molecular-weight form of GCase as a result of increased glycosylation (Fernandes et al., 2016). GCase activity was not assessed in this study, and there was no accumulation of the GCase substrate GlcCer (Fernandes et al., 2016). In another study using a different set of *n* = 3 GBA N370S iPSC differentiated to dopaminergic neurons, a significant decrease in GCase protein was measured in the mutation cell lines compared to controls (Yang et al., 2020). This was associated with a significant ~50% decrease in GCase activity as measured using the 4MUG probe (Yang et al., 2020). A third study employing different heterozygous GBA N370S iPSC lines differentiated to dopaminergic neurons showed an ~25% decrease in GCase activity with the 4MUG probe in the mutation lines compared to controls, and this was a result of decreased protein levels (Schöndorf et al., 2014). In this study, there was also an accumulation of GlcCer substrates (Schöndorf et al., 2014). Reasons for the differences in GCase deficits between the current study and the above could be the use of the PFB-FDGlu probe to measure GCase activity in live cells, as opposed to the use of the 4MUG probe to measure GCase activity in lysed cells. Variation in GCase activity between iPSC lines can also be high, and larger sample sizes are likely required for definitive results. However, all the above studies also used iPSC differentiated into dopaminergic neurons. Alpha-synuclein pathology is not restricted to dopaminergic neurons in PD. However, there may be neural cell-type-specific factors that influence GCase activity. This is potentially highlighted further by a study showing that GCase activity was reduced in dopaminergic neurons differentiated from *n* = 2 PD patients with the LRRK2 G2019S mutation. In this study, there was an ~40% reduction in live cell GCase activity measured with the PFB-FDGlu probe (Ysselstein et al., 2019). Interestingly, there was also a reduction in GCase activity in the patient fibroblasts from which the iPSC was differentiated. However, this was less dramatic, leading to suggestions that GCase activity differences may be potentiated in dopaminergic neurons, potentially due to enhanced oxidative stress in this neural cell type due to dopamine oxidation (Ysselstein et al., 2019). This may explain why less of an effect was observed in the current study using non-dopaminergic LRRK2 G2019S cells, albeit derived from different patients, and the PFB-FDGlu probe that had no correlation between GCase activity in NSCs and differentiated neurons was observed in the current study, which also suggests that differentiation to different cell types can affect GCase activity. Finally, given that neither the LRRK2 G2019S nor GCase N370S mutations are fully penetrant, it is not surprising to find heterogeneity across patient lines, and the ongoing study of additional lines will help to clarify the extent of heterogeneity of these important PD mutations. Efforts to better understand the interplay and modifiers of these enzymes in order to stratify patients for clinical trials are also ongoing.

In summary, we optimized a live cell imaging-based assay for lysosomal GCase activity that can be used to further explore and understand PD phenotypes in differentiated cell models, as well as for screening potential modifying compounds of GCase activity.

## Data availability statement

The original contributions presented in the study are included in the article/Supplementary material, further inquiries can be directed to the corresponding author.

## Author contributions

AL-G: experimental work, data analysis, figure preparation, and manuscript drafting. SZ and SK: experimental work, data analysis, and figure preparation. LH: experimental work and data analysis. WK and GH: manuscript drafting and editing and experimental design. ND: conceived the idea, experimental design, funding, data analysis, figure preparation, and manuscript drafting and editing. All authors contributed to the article and approved the submitted version.
