# Supplementary material for: Live cell in situ lysosomal GCase activity correlates to alpha-synuclein levels in human differentiated neurons with LRRK2 and GBA1 mutations
Source: Front Cell Neurosci. 2023 Oct 16;17:1229213. doi: 10.3389/fncel.2023.1229213 (PMC10613732; doi:10.3389/fncel.2023.1229213)
Supplement: Supplementary file 2 [file Data_Sheet_1.PDF]

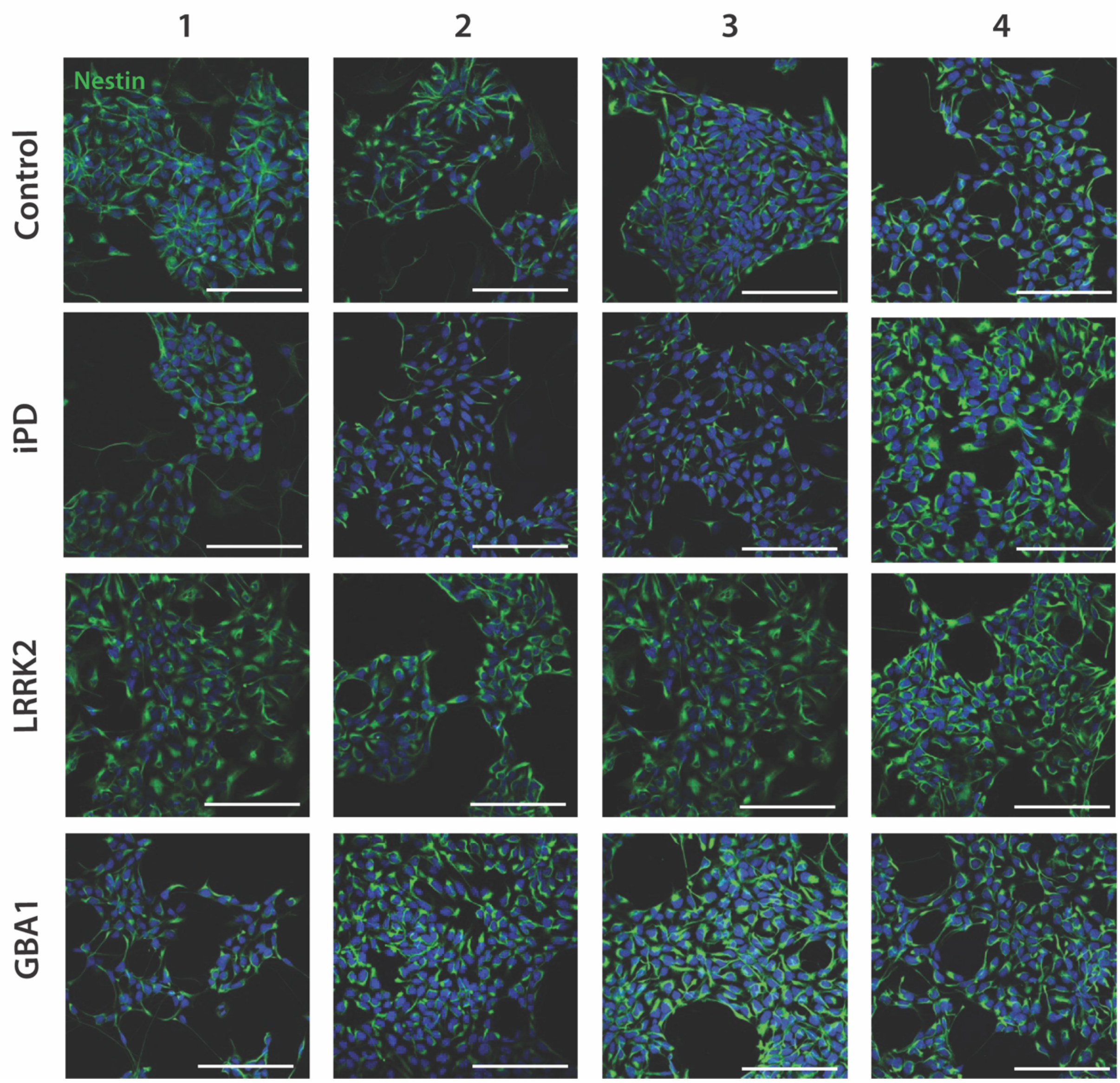

Figure S1: **Representative images of Nestin stained Neural stem cells.** Neural stem cells were differentiated from all iPSC lines and stained with the NSC marker nestin (in green). All lines showed > 90% nestin positive staining by manual counting. Scale bar in white = 100 µm.

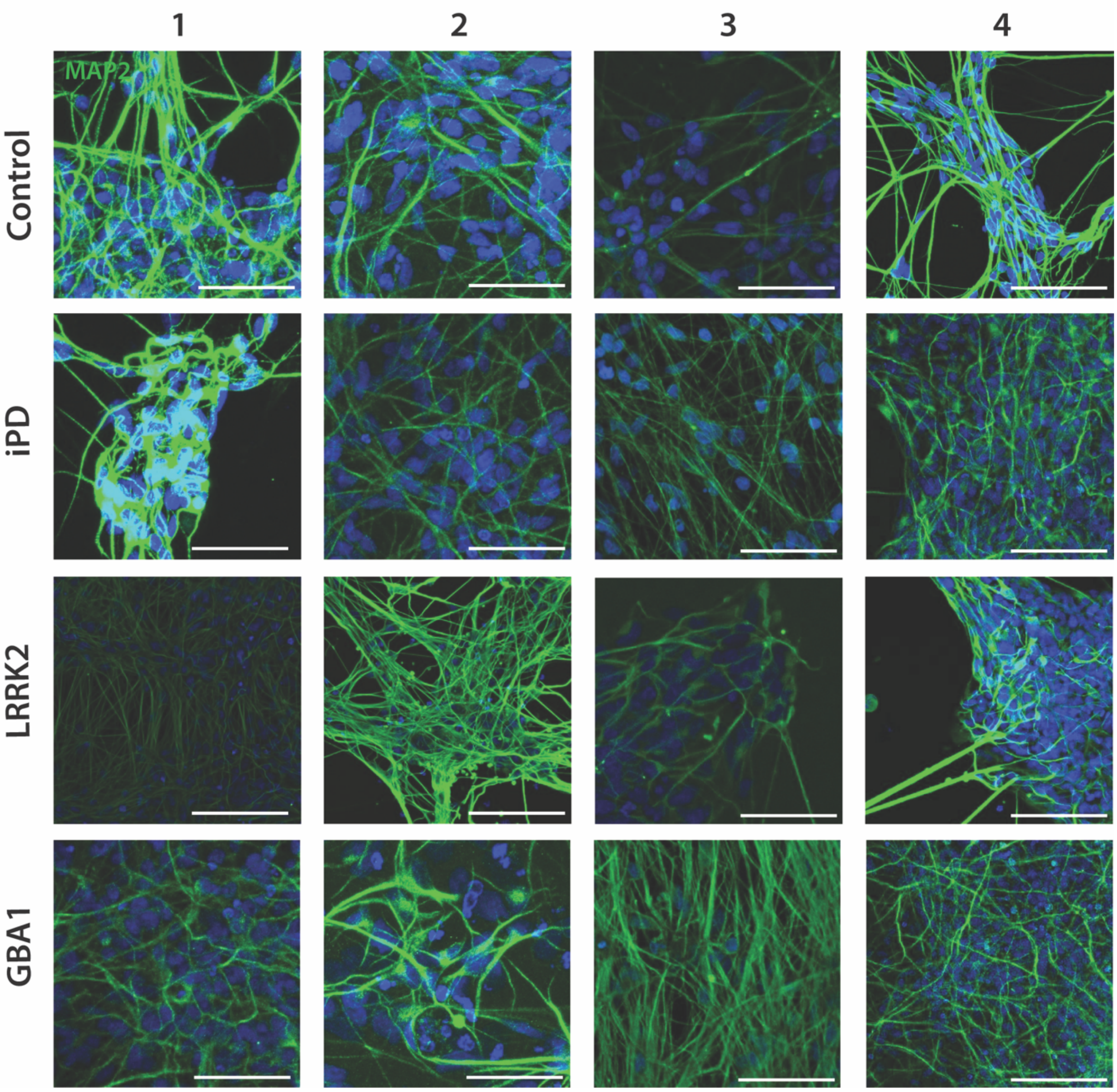

Figure S1: **Representative images of MAP2 stained Neurons.** Neural stem cells were further differentiated to neurons and stained with the neuron marker MAP2 (in green). All lines showed > 90% MAP2 positive staining by manual counting. Scale bar in white = 50 µm
